# Gedächtnisambulanzen in Deutschland – strukturell-organisatorische Voraussetzungen und Aufgabenfelder

**DOI:** 10.1007/s00115-020-01007-7

**Published:** 2020-10-06

**Authors:** Lucrezia Hausner, Lutz Frölich, Christine A. F. von Arnim, Jens Bohlken, Richard Dodel, Markus Otto, Michael Rapp, Jörg Schulz, Tilmann Supprian, M. Axel Wollmer, Frank Jessen, Deutsches Netzwerk Gedächtnisambulanzen

**Affiliations:** 1 Universität Heidelberg, Abteilung Gerontopsychiatrie, Zentralinstitut für Seelische Gesundheit Mannheim, Quadrat I 5, 68159 Mannheim, Deutschland; 2 Abteilung für Geriatrie, Universitätsmedizin Göttingen, Göttingen, Deutschland; 3 Referat Demenz, Berufsverband Deutscher Nervenärzte e. V. (BVDN), Krefeld, Deutschland; 4 Medizinische Fakultät, Gastwissenschaftler Institut für Sozialmedizin, Arbeitsmedizin und Public Health (ISAP), Universität Leipzig, Leipzig, Deutschland; 5 Lehrstuhl für Geriatrie, Geriatriezentrum Haus Berge, Universität Duisburg-Essen, Essen, Deutschland; 6 Institut für Neurologie, Universitätsklinikum Ulm, Ulm, Deutschland; 7 St. Hedwig Krankenhaus, Klinik für Psychiatrie und Psychotherapie, Gerontopsychiatrisches Zentrum, Psychiatrische Universitätsklinik der Charité, Berlin, Deutschland; 8 Klinik für Neurologie, Universitätsklinikum RWTH Aachen, Pauwelsstraße 30, 52074 Aachen, Deutschland; 9 LVR Klinikum Düsseldorf, Abteilung Gerontopsychiatrie, Heinrich-Heine-Universität Düsseldorf, Düsseldorf, Deutschland; 10 Klinik für Psychiatrie und Psychotherapie – Klinik für Gerontopsychiatrie, Asklepios Klinik Nord – Ochsenzoll, Hamburg, Deutschland; 11 Medizinische Fakultät, Klinik und Poliklinik für Psychiatrie und Psychotherapie, Uniklinik Köln, Kerpener Str. 62, 50924 Köln, Deutschland; 12 Deutsches Zentrum für Neurodegenerative Erkrankungen Bonn (DZNE), Sigmund-Freud-Str. 27, 53127 Bonn, Deutschland; 13 Referat Prävention psychischer Erkrankungen, Deutsche Gesellschaft für Psychiatrie und Psychotherapie, Psychosomatik und Nervenheilkunde e. V. (DGPPN), Berlin, Deutschland

**Keywords:** Demenz, Gedächtnisambulanz, Struktur, MCI, Harmonisierung, Dementia, Memory clinic, Structure, Harmonization, MCI

## Abstract

**Hintergrund:**

Gedächtnisambulanzen (GA) sind auf (Differenzial‑)Diagnostik, Therapie, Aufklärung, Management und Beratung von kognitiven Störungen im Alter und deren Risikostadien spezialisierte Einrichtungen. In der Praxis haben sie sehr unterschiedliche Organisationsformen. Aufgrund der wachsenden diagnostischen Möglichkeiten bei neurodegenerativen Erkrankungen, dem steigenden Bedarf an Früherkennung und Prädiktion sowie absehbaren neuen diagnostischen Verfahren und krankheitsmodifizierenden Therapien ist eine Vereinheitlichung der strukturellen Voraussetzungen und Aufgabenbereiche für GA sinnvoll.

**Ziel der Arbeit:**

Der Artikel macht Vorschläge für strukturelle und organisatorische Voraussetzungen, Aufgaben sowie einheitliche Arbeitsweisen von GA in Deutschland.

**Methoden:**

Expertenkonsens von Psychiatern, Neurologen und Geriatern aus universitären und außeruniversitären Einrichtungen.

**Ergebnisse:**

Gedächtnisambulanzen sollen den Facharztstandard für Psychiatrie und/oder Neurologie vorhalten und sich in ihrer Arbeitsweise bez. (Differenzial‑)Diagnostik und Therapie von Demenzen eng an der S3-Leitlinie (S3LL-)Demenz orientieren. In Bezug auf Früherkennung und Prädiktion neurodegenerativer Erkrankungen gehen sie über die S3LL-Demenz hinaus. So werden leichte kognitive Störungen (MCI) als Risiko- oder auch Prodromalstadien neurodegenerativer Demenzen verstanden und Biomarker regelhaft für eine ätiologische (Früh- und Differenzial‑)Diagnostik eingesetzt. Es soll eine enge Vernetzung mit den diagnostischen Fachdisziplinen bestehen. Ferner sollen sie Beratung zu sozialen und rechtlichen Fragen sowie Angehörigenberatung anbieten. Aktuelle Erkenntnisse aus der Forschung sollen durch sie frühzeitig in die Versorgung integriert werden. GA sind damit regionale Expertenzentren.

**Diskussion:**

Gedächtnisambulanzen implementieren den evidenzbasierten Standard in Diagnostik und Therapie in die klinische Versorgung von Patienten mit kognitiven Störungen im Alter. Zusätzlich führen sie diagnostische und therapeutische Innovationen in die Versorgung dieser Patienten ein. Ihre Rolle in der Regelversorgung muss gestärkt werden, wobei auch Finanzierungsfragen geklärt werden müssen, da die derzeitigen Finanzierungsmodelle in der Regel nicht kostendeckend sind.

## Abstract (en)

**Background:**

Memory clinics (MC) are institutions specialized in the (differential) diagnostics, treatment, education, management and counseling of diseases related to dementia and their risk stages. In Germany, they have a variety of different organizational forms. Due to the growing diagnostic options in neurodegenerative diseases, the increasing demand for early detection and prediction as well as foreseeable new diagnostic procedures and disease-modifying treatment, it is important to standardize the structural prerequisites and areas of responsibility of MC.

**Objective:**

The article proposes structural and organizational requirements and procedures and a harmonized mode of operation for MC in Germany.

**Method:**

Expert consensus of psychiatrists, neurologists and geriatricians from academic and nonacademic institutions.

**Results:**

The MC should provide the specialist standards of psychiatry and/or neurology. They need to implement the recommendations of the national guidelines on dementia (S3LL) with respect to the (differential) diagnostics and treatment of dementia. With respect to the early detection and prediction of neurodegenerative disorders, they extend beyond the current German guideline standards. In MC, mild cognitive impairment (MCI) is understood as an at-risk or prodromal stage of diseases related to dementia and biomarkers are consistently applied for etiological (early and differential) diagnostics. There is a requirement for close interaction with specialized diagnostic disciplines. Furthermore, MC should also offer comprehensive advice on social and legal issues and provide caregiver support. They should integrate current knowledge from research into care and serve as regional expert centers.

**Conclusion:**

The MC should implement evidence-based standards in diagnostics and treatment and introduce innovations in the care of patients with cognitive disorders and at-risk and prodromal stages. Their role in the German healthcare system must be strengthened. Sufficient and sustained funding needs to be established, since current reimbursement does not cover costs.

## Einleitung

Gedächtnisambulanzen (GA) sind spezialisierte medizinische Einrichtungen für die Diagnostik und Differenzialdiagnostik, Behandlung und Beratung von Patienten mit kognitiven Störungen im höheren Alter. Es existieren mindestens 200 GA in Deutschland. Es sind organisatorisch sehr heterogene Institutionen, u. a. universitäre Forschungsambulanzen, psychiatrische Institutsambulanzen, medizinische Versorgungszentren oder Schwerpunktpraxen, die sich daher auch in ihrer Arbeitsweise unterscheiden [1]. Zusätzlich haben GA Fortbildungsfunktionen für medizinische Berufe und eine Informations- und Aufklärungsfunktion für die Allgemeinbevölkerung. Ein Konsens über strukturelle Mindeststandards sowie diagnostische und therapeutische Angebote von GA existiert noch nicht. Universitäre GA verfolgen neben der Patientenversorgung häufig Implementierungs- und klinische Forschung, während z. B. Versorgungskliniken mit GA oftmals eine längerfristige Betreuung von Demenzerkrankten anbieten. Die gemeinsame Zielgruppe von GA sind sowohl Patienten mit Demenz als auch Patienten in Risiko- und Frühstadien neurodegenerativer Erkrankungen. Im Rahmen der Differenzialdiagnostik werden auch Patienten mit nichtneurodegenerativen Erkrankungen versorgt, sofern kognitive Störungen das klinische Bild bestimmen.

Mehrere Entwicklungen werden in naher Zukunft die Anforderungen an GA erhöhen:

1. Die Zunahme von kognitiven Störungen im Alter aufgrund des demographischen Wandels.
2. Eine erhöhte „awareness“ gegenüber altersbezogenen kognitiven Störungen [19], der eine Einstellungsänderung in der Bevölkerung und bei den Behandlern zur Diagnostik und Therapie kognitiver Störungen im Alter zugrunde liegt [12].
3. Die Erkenntnis, dass die leichte kognitive Störung (MCI) Ausdruck einer beginnenden neurodegenerativen Erkrankung sein kann [4, 32], verbunden mit verbesserten diagnostischen Möglichkeiten. Hierbei ist die ätiologische Zuordnung der MCI-Genese mittels Biomarkern und die konsekutive prognostische Fragestellungen zu einem zentralen Punkt in der Versorgung [22, 33] geworden.
4. Ferner werden erwartete spezifische Therapien, wie beispielsweise monoklonale Antikörper, eine bessere biologische Charakterisierung der Patienten mit Gedächtnisstörungen erfordern [6].

Ziel des vorliegenden Artikels ist es, strukturelle und arbeitsorganisatorische Mindeststandards für GA in Deutschland zu definieren und deren Umsetzung, z. B. im Rahmen einer standardisierten Qualitätssicherung, vorzuschlagen. Hierzu wurde ein Expertenkonsensus von Vertretern psychiatrischer, neurologischer und geriatrischer universitärer und außeruniversitärer Zentren erarbeitet. Das Papier wurde ferner von dem sich aktuell in Gründung befindlichen Deutschen Netzwerk Gedächtnisambulanzen konsentiert. Dieses Netzwerk ist eine Vereinigung der GA in Deutschland mit dem Zweck, einheitliche Vorgehensweisen zu entwickeln und die Vernetzung untereinander zu fördern.

## Personelle Ausstattung von Gedächtnisambulanzen

**Figure 1:**
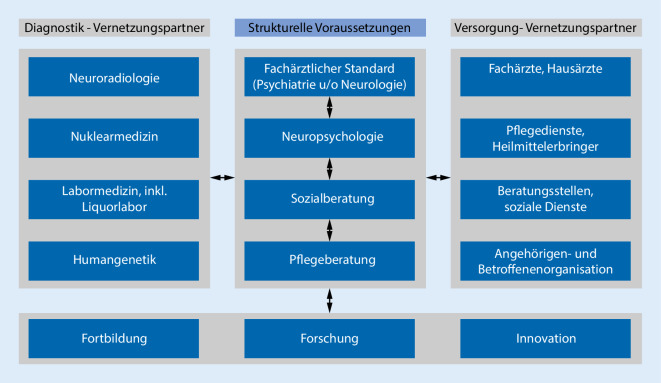

Eine zentrale Kompetenz einer Geächtnisambulanz (GA) ist die (Früh‑)Diagnostik und die damit verbundene Therapie kognitiver Störungen im Alter. Dafür ist ein multiprofessionelles Team mit folgenden Kompetenzen notwendig: spezialisierter Facharztstandard in Psychiatrie und/oder Neurologie, neuropsychologische (NP) Kompetenz für demenzspezifische NP-Testverfahren und sozialarbeiterische Kompetenz für Versorgungs- und Pflegeberatung. Eine enge Vernetzung mit Kliniken oder Instituten für Neuroradiologie, Nuklearmedizin und klinische Chemie inklusive spezialisiertem Liquorlabor sowie Humangenetik sollte vorhanden sein (Abb. 1).

## Ärztliche Aufgaben (Diagnostik, Diagnosevermittlung und Behandlung, Beurteilung von Einwilligungsfähigkeit, Beratung bez. Fahreignung, klinisches Management)

Das ärztliche Aufgabenspektrum umfasst die Anamnese und Fremdanamnese sowie die systematische neurologisch-psychiatrische und allgemeine klinische Untersuchung des Patienten. Im Rahmen dessen sollen auch motivationale Aspekte der Vorstellung erfasst werden. Die bestehende Medikation soll auf potenziell dyskognitiv wirksame Medikamente überprüft werden. Ärztliche Kernaufgaben sind ferner die Aufklärung über diagnostische Untersuchungen und deren Auswahl, die kontextualisierte Bewertung diagnostischer Befunde und ihre prognostische Beurteilung. Befundinterpretation und Prognoseschätzung haben speziell im Bereich der biomarkerbasierten Frühdiagnostik eine besondere Relevanz. Die Fremdanamnese durch eine informierte Begleitperson dient dem Abgleich von Selbst- und Fremdwahrnehmung bestehender Symptome, deren Verlaufsentwicklung, der Erfassung der Alltagskompetenz einschließlich Einschränkungen in komplexen Aktivitäten (iADL) und der Bewertung von psychischen und Verhaltenssymptomen. Für die systematische Erfassung der Alltagskompetenzen und der psychischen und Verhaltenssymptome können standardisierte Instrumente mit genutzt werden.

Bei Demenzerkrankungen handelt es sich um schwerwiegende Diagnosen mit begrenzten Therapieoptionen und der Prognose einer Pflegebedürftigkeit sowie reduzierten Lebenserwartung. Sie bedürfen einer stadiengerechten Aufklärung. Die Diagnosestellung und Initiierung einer antidementiven Therapie sowie die Verordnung nichtmedikamentöser kognitiver Therapien (z. B. kognitiv-funktionelle Ergotherapie) erfolgt durch den Arzt der Gedächtnisambulanz (GA). Weitere Behandlungsempfehlungen (z. B. Risikofaktormanagement) sollen gemeinsam mit dem Hausarzt und anderen Fachärzten umgesetzt werden. Der GA-Arzt klärt die Patienten und Angehörigen auf und berät auch über weitere Unterstützungsangebote für Patienten und Angehörige (z. B. Psychotherapie). Es soll ein individuell angepasstes Behandlungs- und Betreuungskonzept entworfen werden, welches entsprechend der Krankheitsentwicklung über die Zeit modifiziert werden muss. Eine mittelfristige Weiterbetreuung durch die GA für den Fall einer kognitiven Zustandsverschlechterung sollte gebahnt werden. Patienten, die im Rahmen der Frühdiagnostik vorstellig werden, müssen vor der Durchführung biomarkerbasierter diagnostischer Maßnahmen über deren Implikationen beraten werden. Hierbei ist insbesondere über die Möglichkeiten und Grenzen der Abschätzung des individuellen Progressionsrisikos zu beraten [31]. Die Patienten sind über das Recht auf Wissen vs. das Recht auf Nichtwissen zu informieren.

GAs sollten eine leitliniengerechte antidementive Pharmakotherapie unter Berücksichtigung von Komorbiditäten initiieren, steuern und gegebenenfalls anpassen. Diese soll auch immer nichtmedikamentöse Therapieoptionen umfassen [10, 19]. Kognitive Trainings- und Stimulationsverfahren können insbesondere in Frühstadien angeboten werden. Psychische Symptome und Verhaltenssymptome sind in allen Erkrankungsstadien gesondert zu berücksichtigen. Sie sollen bevorzugt durch nichtmedikamentöse Therapieverfahren behandelt werden. Darüber hinaus ist eine leitlinienbasierte Anwendung von Psychopharmaka zu erwägen, falls mit nichtpharmakologischen Behandlungen keine ausreichende Besserung erreicht werden kann. Es ist möglich, Patienten mit leichter kognitiver Störung (MCI) oder leichter Demenz psychotherapeutisch zu betreuen, was in der Praxis aber kaum umgesetzt wird. Therapeutische Ziele können die Reduktion von Depression und Angst sein oder die Verbesserung der Anpassung an die Demenzerkrankung und Erhöhung der Lebensqualität [18]. Bei Bedarf sollte ein Psychotherapeut vermittelt werden. Eine psychosoziale Therapie kann die Belastungen durch die Diagnosestellung und zunehmende Symptome im Verlauf sowie interaktionelle Konflikte reduzieren [18]. Eine enge Zusammenarbeit von Neurologie und Psychiatrie insbesondere im Hinblick auf komorbide Depressionen, Bewegungsstörungen und vaskuläre Erkrankungen ist sinnvoll.

Kognitive Kompetenzen mit hoher Alltagsrelevanz und auch rechtlichen Konsequenzen sind Einwilligungsfähigkeit, Geschäftsfähigkeit und Fahrtauglichkeit. Die Beurteilung der Einwilligungsfähigkeit bewertet die Fähigkeit, Bedeutung, Tragweite und Risiken medizinischer Maßnahmen zu erfassen und den Willen diesbezüglich bestimmen und formulieren zu können. Die Beurteilung von Einwilligungsfähigkeit erfolgt anlassbezogen z. B. in Bezug auf die jeweilige geplante medizinische Maßnahme. Die Beurteilung der Geschäftsfähigkeit erfolgt zustandsbezogen und bewertet die Fähigkeit, Rechtsgeschäfte selbstständig vollwirksam vorzunehmen. Ihre Beurteilung gehört jedoch nicht zur klinischen Routineversorgung von GA, sondern erfolgt vor allem im Rahmen von Gutachten. Eine Demenz beeinträchtigt beide Fähigkeiten zunehmend und hebt sie letztlich auf. Daher müssen GA-Ärzte für eine korrekte Beurteilung besonders geschult sein. Grundsätzlich ist im Diagnostikprozess dies mit Patienten und Angehörigen zu thematisieren und die Einrichtung einer Vorsorgevollmacht oder Betreuungsverfügung bei bestehender Einwilligungsfähigkeit zu empfehlen. Die Einwilligungsfähigkeit in Bezug auf eine medizinische Maßnahme soll immer dokumentiert werden. Bei aufgehobener Einwilligungsfähigkeit sollen Bevollmächtigte oder bestellte rechtliche Vertreter (Betreuer) herangezogen bzw. sollen sie bei Gericht vorgeschlagen werden, wenn diese noch nicht existieren.

Fragen zur Fahreignung sind häufig ein sensibles Thema in der Versorgung und Beratung von Patienten und Angehörigen einer GA, da diese entweder bei Diagnosestellung bereits nicht mehr gegeben ist oder im Behandlungsverlauf verlorengehen wird. Deshalb ist es die Pflicht des GA-Arztes, eine verkehrsrelevante Symptomatik zu erfragen. Demenz und MCI beeinträchtigen in variablem Ausmaß die Fahrtauglichkeit und addieren sich zu anderen altersbedingten Beeinträchtigungen. Daher wird in Begutachtungsleitlinien zur Kraftfahreignung [29] und der Fahrerlaubnisverordnung [11] kein grundsätzliches Fahrverbot bei MCI oder leichter Demenz gefordert. Bestimmte Demenzätiologien beeinträchtigen mit hoher Wahrscheinlichkeit die Fahrtauglichkeit (z. B. Impulsivität bei frontotemporaler Demenz, Vigilanzschwankungen bei Parkinson- oder Lewy-Körperchen-Demenz), worüber Patienten und Angehörige gesondert aufgeklärt werden sollen. Bezogen auf das Demenzstadium ist die individuelle Symptomheterogenität zu berücksichtigen. Über eine vorliegende oder im Erkrankungsverlauf auftretende Beeinträchtigung der Fahrtauglichkeit soll der GA-Arzt die Patienten bei Diagnosestellung aufklären und dies schriftlich dokumentieren. Die Fahrtauglichkeit sollte mindestens ab dem Stadium einer leichten Demenz überprüft werden. Angehörige sollten über Frühwarnzeichen für verminderte Fahreignung informiert werden (u. a. Desorientiertheit an Kreuzungen, verzögerte und unsichere Reaktionen, Bagatellschäden) und gegebenenfalls auf eine Fahrverhaltensprobe hinwirken. Detaillierte Empfehlungen zum ärztlichen Vorgehen zur Beurteilung der Fahrtauglichkeit im Rahmen von Demenzerkrankungen sind publiziert [14]. Relevante gesetzliche Bestimmungen sind in der Fahrerlaubnisverordnung hinterlegt. Im Patientenrechtegesetz ist die Verpflichtung zur Aufklärung über Krankheiten und Therapien geregelt [11], die auch die Aufklärung über Fahreignung umfasst.

## Neuropsychologie

Eine differenzierte NP-Diagnostik dient der Objektivierung und Quantifizierung kognitiver Leistungsdefizite, insbesondere bei Patienten mit leichteren Beeinträchtigungen [2]. Durch sie können die kognitiven Leistungsdefizite den spezifischen kognitiven Domänen (z. B. Auffassung, Gedächtnis) zugordnet werden. Dieses Profil der NP-Defizite unterstützt die Differenzialdiagnostik. Die NP-Testung dient auch der Beurteilung der Alltagskompetenz. Im deutschsprachigen Raum hat sich die CERADplus-Testbatterie als quasi Standard der NP-Testung für Demenzen durchgesetzt. Für diese sind normative Daten für den deutschsprachigen Raum verfügbar. Es existiert eine Vielzahl validierter NP-Einzeltests, welche prinzipiell als Alternative für die Subtests der CERADplus-Testbatterie eingesetzt werden können. Bei ihrer Auswahl sollte darauf geachtet werden, dass alle Domänen der CERADplus-Testbatterie abgedeckt sind [5]. Zur Diagnostik von Prädemenzstadien und zur erweiterten Differenzialdiagnostik sollen weitere spezifische NP-Testverfahren vorgehalten werden.

## Soziale und rechtliche Beratung

Patienten und Angehörigen sollte Sozialberatung in der GA angeboten werden. Hierzu gehört die Beratung hinsichtlich der Gewährung von Leistungen der Kranken- und Pflegeversicherung, zur Frage der Schwerbehinderung sowie zu den Themen Betreuung, Betreuungsverfügung, Vollmacht und Patientenverfügung. Bei noch berufstätigen Patienten sind Fragen zur Berentung ein wichtiges Thema. Die Beratung kann durch Sozialarbeiter oder andere speziell geschulte Mitarbeiter erfolgen. Über eine mögliche Forschungsverfügung muss ein Arzt beraten.

Von besonderer Bedeutung ist die Angehörigenberatung und -schulung. Innovative Versorgungsansätze wie individualisiertes Fallmanagement und aufsuchende Unterstützung und Behandlung sollten ausgeschöpft werden. Grundsätzlich sollte die Therapie mit anderen Beteiligten (Hausärzte, Fachärzte, Pflegedienste, Betreuern etc.) abgestimmt werden. GA sollten Informationsveranstaltungen und Schulungen anbieten können. Eine Lebensumfeldberatung, auch in Form von Hausbesuchen, sollte von Ergotherapeuten durchgeführt werden.

Anlaufstellen für eine Beratung und Schulung zur Pflege sollen empfohlen werden. Dies ermutigt und motiviert Angehörige und Patienten mit Demenz, Pflegestrukturen in Anspruch zu nehmen, was die Versorgung und Lebensqualität dieser erhöht.

## Partner für die Diagnostik

### Neuroradiologie

Eine Kooperation mit einer neuroradiologischen Abteilung oder Klinik ist erforderlich. Gemeinsame Standards der Untersuchung und Befundung sollen definiert werden. Aufgrund der höheren Sensitivität ist eine zerebrale Magnetresonanztomographie (MRT) ohne Kontrastmittel für die Diagnostik vieler zerebraler Pathologien bei neurodegenerativen und vaskulären Erkrankungen gegenüber einer Computertomographie (CT) zu bevorzugen. Die strukturelle Bildgebung dient dem Ausschluss potenziell reversibler zerebraler Ursachen kognitiver Störungen (z. B. Normaldruckhydrozephalus, Meningeom) sowie fokaler intrazerebraler Pathologien und vaskulärer Hirnveränderungen. Hierbei wird aus den Bildern auch differenzialdiagnostische Information zu primären Demenzerkrankungen abgeleitet, z. B. regionale Atrophien bei frontotemporaler Demenz. Regionale Atrophien, z. B. des medialen Temporallappens, das Ausmaß der Mikroangiopathie und die parietale Hirnatrophie sollen über visuelle Ratingskalen (z. B. Scheltens-Skala, Fazekas-Skala, Koedam-Skala) bewertet werden. Automatisierte quantitative Auswertungsalgorithmen für die globale Atrophie, fokale Atrophien und Gefäßerkrankungen sind alternativ verfügbar, müssen aber bez. ihrer Validität im Rahmen der jeweiligen lokalen apparativen Voraussetzungen gesichert sein. Bildgebende Befunde sollen dabei stets kontextualisiert interpretiert werden.

### Nuklearmedizin

Verfahren der Nuklearmedizin gehören zwar nicht zur Basisdiagnostik von Demenzerkrankungen, können aber bei verschiedenen Fragestellungen zur Anwendung kommen. In der Differenzialdiagnostik von Demenzerkrankungen dienen das [99mTc]HMPAO- Single Photon Emission Computed Tomography (SPECT) und das 18F-FDG-Positronen-Emissions-Tomographie (PET) der Messung der Hirnperfusion bzw. des Glukosemetabolismus. Das [123I]FP-CIT-SPECT ist für die Diagnostik neurodegenerativer Erkrankungen mit dopaminerger Beteiligung indiziert. Über die Empfehlungen der S3-Leitlinien Demenz (S3LL) [10] hinaus ist ihr Einsatz auch bei MCI sinnvoll, um Hinweise auf die Ätiologie der Beeinträchtigung zu erhalten.

Die Amyloid-PET unter Verwendung 18Fluor-markierter Tracer erlaubt den Nachweis der intrazerebralen Amyloidpathologie [23] und ist für die (Früh‑)Diagnostik der Alzheimer-Krankheit (Nachweis einer Amyloidpathologie) in Deutschland zugelassen. Sie kann als Amyloidbiomarker auch bei Fragen im Rahmen von MCI eingesetzt werden. Internationale Empfehlungen zum klinischen Einsatz des Amyloid-PET wurden publiziert [16]. Alle Befunde müssen im klinischen Kontext beurteilt werden. Die gesetzlichen Krankenversicherungen erstatten jedoch die Kosten eines Amyloid-PET im Regelfall nicht.

### Labormedizin

Im Rahmen der ätiologischen Diagnostik ist eine differenzierte serologische und biochemische Blutuntersuchung primär zum Ausschluss sekundärer Demenzursachen entsprechend der S3-LL-Demenz [10] erforderlich. Blutbasierte Biomarker für neurodegenerative Erkrankungen sind in dynamischer Entwicklung, aber für den klinischen Einsatz noch nicht ausreichend validiert.

Die Untersuchung des Liquor cerebrospinalis („cerebrospinal fluid“, CSF) dient dem Ausschluss nichtneurodegenerativer, z. B. entzündlicher Ätiologien kognitiver Störungen sowie gleichzeitig der Erfassung spezifischer Biomarker für neurodegenerative Erkrankungen. Sie soll standardisiert durchgeführt werden, inklusive einem definierten präanalytischen Verarbeitungsalgorithmus [9]. Die CSF-Analysen sollen in einem Labor durchgeführt werden, das Erfahrung in der Untersuchung von Biomarkern für neurodegenerative Erkrankungen besitzt. Die bestehende Varianz zwischen Laboren kann durch vollautomatisierte Analysesysteme reduziert werden. Im CSF ist anhand von vermindertem Amyloid-β-1–42 bzw. erniedrigter Amyloid-β-42/40-Ratio zusammen mit einer erhöhten Konzentration von Gesamt-Tau (t-Tau) und/oder hyperphosphoryliertem Tau (p-Tau) der In-vivo-Nachweis der Alzheimer-Pathologie in allen symptomatischen Erkrankungsstadien möglich [15]. Prognosemodelle für die Entwicklung einer Demenz bei Patienten mit MCI mithilfe der genannten Biomarker sind publiziert und eine individuelle Risikoabschätzung wird zunehmend möglich [31, 33]. Auch für andere neurodegenerative Demenzen stehen spezifische CSF-Marker zur Verfügung (z. B. Protein 14-3‑3, RT-QuIC, onkoneuronale Antikörper). CSF-Biomarker sind, ähnlich wie die Amyloid-PET-Bildgebung, Bestandteil aktueller Forschungskriterien zur Diagnose der Alzheimer-Krankheit. Zukünftig werden wahrscheinlich weitere Marker, wie z. B. Neurofilamentleichtketten (NFL) im Blut und Liquor als Teil des neuronalen Stütz- und Zytoskeletts und YKL-40, CHIT‑1 als Marker der Neuroinflammation sowie SNAP-25 und β‑Synuklein als Marker für einen Synapsenschaden in die Liquordiagnostik Eingang finden. Diese sind aber für einen Einsatz in der breitgestreuten Routine noch nicht ausreichend validiert.

### Genetische Diagnostik

Beim Verdacht auf eine genetisch bedingte Demenzerkrankung ist die Indikation für eine molekulargenetische Diagnostik grundsätzlich gegeben. Diese diagnostische Untersuchung mit vorangehender Beratung kann bei symptomatischen Patienten durch jeden GA-Arzt vorgenommen werden. Patienten und Angehörige sind bei klinischem Verdacht auf eine krankheitsauslösende Mutation vor der gendiagnostischen Untersuchung durch den Arzt zu beraten. Prädiktive gendiagnostische Untersuchungen können bei nichterkrankten Personen nach Beratung gemäß Gendiagnostikgesetz auf Wunsch durch einen Humangenetiker oder durch einen dafür zertifizierten Facharzt initiiert werden. Die Kooperation mit einem Institut für Humangenetik ist empfehlenswert.

### Forschung und klinische Studien

Ein über Versorgungsaufgaben hinausgehender Schwerpunkt, insbesondere universitärer GA, ist die klinische Forschung mit Implementierungsforschung und klinischen Studien, auch nach dem Arzneimittelgesetz (AMG). Für Patienten und Angehörige kann die Teilnahme an klinischen Studien auch mit sich in Entwicklung befindlichen und noch nicht zugelassenen Medikamenten oder anderen Interventionen eine ergänzende Option zur Behandlung darstellen [26]. Zunehmend werden auch digitale Angebote zur Diagnostik oder zum Training bei Demenzerkrankungen wissenschaftlich evaluiert.

## Perspektiven und Konsequenzen

Bis zum Jahr 2050 wird mit einer Zunahme der weltweiten Demenzprävalenz auf 131 Mio. Erkrankte gerechnet [19]. Für Deutschland bedeutet das einen Anstieg betroffener Personen von derzeit 1,7 Mio. auf über 3 Mio. [7]. Hinzukommen werden die Personen mit MCI, deren Prävalenz (16,6 % für über 65-Jährige) ebenfalls stark altersabhängig zunimmt [25]. Bereits jetzt stellt die medizinische Versorgung von Demenzpatienten eine große Herausforderung dar. Gleichzeitig sind in der klinischen Forschung große Fortschritte zu verzeichnen, wie neue Krankheitskonzepte, Früherkennung und Demenzprädiktion, die Biomarkerentwicklung inklusive blutbasierter Biomarker und hoffentlich zukünftige neue Therapieansätze. Bei der Implementierung dieser Erkenntnisse ergeben sich zahlreiche Herausforderungen, wie methodische Validierung, Übertrag- und Generalisierbarkeit, ethische und rechtliche Fragen sowie erforderliche Regelungen in Bezug auf Finanzierung und Zugänglichkeit. Zusätzlich nehmen die Möglichkeiten der Differenzierung von Demenzerkrankungen in vivo weiter zu. So werden z. B. auf der Basis der neuropathologischen Erkenntnisse neue Demenzformen definiert (z. B. „limbic-predominant age-related TDP-43 encephalopathy“, LATE; [24]). Auch diese Weiterentwicklungen müssen von GA berücksichtigt werden.

Die Zahl von Patienten, die in GA vorstellig werden, ist derzeit noch gering im Vergleich zur Gesamtzahl der Demenz- und MCI-Patienten, auch weil es zu wenig GA in Deutschland gibt und die Kenntnisse über Aufgabenfelder und Kompetenzen der GA bei Zuweisern wie Haus- und Nervenärzten und in der Allgemeinbevölkerung begrenzt sind [3]. Ferner existiert keine spezifische Finanzierung der GA. Da, wie dargestellt, die zeitliche Anforderung bei der Diagnose und der ärztlichen, psychologischen und therapeutischen Betreuung hoch ist und zahlreiche Zusatzuntersuchungen für eine sichere Diagnose notwendig sind, ist diese in der Regel nicht ausreichend und wird dem Bedarf nicht gerecht. Weil aber viele Patienten bereits im Stadium des MCI oder der leichten Demenz differenzierte medizinische Hilfe suchen und ihr Anteil am gesamten Patientenkollektiv weiter zunehmen wird [12], ist es dringend notwendig, GA zu stärken und auszubauen und einheitliche Konzepte für die GA-spezifische Versorgung sowie die genannten Fragen und Herausforderungen zu entwickeln. Dies wird die im Versorgungssystem einmalige spezifische Expertise der GA für eine Vielzahl der Patienten besser nutzbar machen.

Inhaltlich ist es wichtig, dass GA die Empfehlungen der nationalen S3LL-Demenz [10] konsequent umsetzen. Diese geben für Diagnostik und Therapie der wichtigsten Demenzformen konsentierte Algorithmen vor, die in der Praxis oft unzureichend umgesetzt werden [20]. Dies ist wahrscheinlich auch auf konkurrierende Leitlinien, z. B. im hausärztlichen Bereich (Leitlinie der Deutsche Gesellschaft für Allgemeinmedizin und Familienmedizin [8]), zurückzuführen. Es gibt zum aktuellen Zeitpunkt in Deutschland noch keinen konsentierten Diagnostik- und Behandlungsalgorithmus für Patienten in Risiko- oder Prodromalstadien von Demenzerkrankungen, z. B. MCI. Mit der Implementierung von Diagnose- und Prädiktionsalgorithmen bei Patienten mit MCI unter Nutzung der Biomarker [28] sollen die GA ebenfalls neue wissenschaftliche Evidenzen aufnehmen [17], die bereits weitgehend validiert [33], aber noch nicht in die Handlungsempfehlungen der S3-LL-Demenz [10] eingegangen sind bzw. über diese hinausgehen. Für die neuen ethischen Herausforderungen und Aufklärungserfordernisse dieser Frühdiagnostik [30], z. B. bez. der individualisierten Prognoseschätzung und der Interpretation potenziell widersprüchlicher Biomarkerbefunde [13, 21, 34], müssen Handlungsempfehlungen entwickelt werden.

Die biomarkerbasierte Frühdiagnostik hat erhebliche Relevanz für die Indikation zukünftiger krankheitsmodifizierender Therapien. Ihr Einsatz wird v. a. bei leicht kranken Patienten, d. h. Patienten im Prodromalstadium oder mit leichter Demenz Erfolg versprechend sein [27]. Dafür wird eine differenzierte biomarkerbasierte Diagnostik mit Amyloidnachweis und möglicherweise auch Bestimmung des APOE-Genotyps Voraussetzung sein. Um diese Therapien erfolgreich zu implementieren und in ihrem Verlauf zu überwachen, inklusive der Bewertung von z. B. therapiebezogenen MRT-Veränderungen („amyloid related imaging abnormalities“, ARIA), ist der Expertenstatus der GA erforderlich. Mit der langfristigen Perspektive einer „kognitiven Medizin“ übernehmen GA auch eine Vorreiterrolle in der interdisziplinären Zusammenarbeit, welche sich auf weitere Disziplinen ausdehnen wird. Um diese Perspektive adäquat in der Versorgung von Demenzen umzusetzen, müssen bestehende ältere Konzepte einer integrierten Versorgung aktualisiert und erweitert werden, wie sie im Rahmenkonzept Demenz formuliert sind [30]. Das Deutsche Netzwerk Gedächtnisambulanzen soll hierfür die organisatorische Plattform bilden.

## Fazit für die Praxis

Gedächtnisambulanzen (GA) sollen Expertenzentren vergleichbaren Standards sein für die komplexe stadiengerechte Diagnostik, medikamentöse und nichtmedikamentöse Therapie, Aufklärung und Beratung von Patienten mit kognitiven Störungen im Kontext von Demenzerkrankungen. Dafür bedarf es einer harmonisierten, vergleichbaren Arbeitsweise besonders im diagnostischen Ablauf orientiert an der S3-Leitlinie Demenz (S3LL) – auch erweitert auf die leichte kognitive Störung (MCI) und andere Risikokonstellationen. Die Implementierung von Qualitätsstandards, wissenschaftlichen Innovationen und eine bestmögliche Versorgung von Patienten sollen damit zukünftig gesichert werden. Ihre Rolle in der Regelversorgung muss gestärkt werden, wobei dies auch Finanzierungsfragen einschließt, da die derzeitigen Finanzierungsmodelle oft keine Deckung der Kosten erlauben. Die Schaffung neuer Strukturen der Vernetzung kann hierfür ebenfalls hilfreich sein.
